# Distribution of bacteria in different regions of the small intestine with *Zanthoxylum bungeanum* essential oil supplement in small-tailed Han sheep

**DOI:** 10.3389/fmicb.2022.1062077

**Published:** 2022-12-23

**Authors:** Hailong Zhang, Xia Lang, Yanshu Zhang, Cailian Wang

**Affiliations:** ^1^Key Laboratory of Bovine and Ovine Germplasm and Straw Transfer into Feedstuff in Gansu Province, Institute of Livestock, Grass and Green Agriculture, Gansu Academy of Agricultural Sciences, Lanzhou, China; ^2^College of Animal Science and Technology, Gansu Agricultural University, Lanzhou, China

**Keywords:** ruminant, *Zanthoxylum bungeanum* essential oil, gut microbiota, 16s rRNA gene sequencing, LEfSe analysis

## Abstract

*Zanthoxylum bungeanum* essential oil (EOZB) as an extract of *Zanthoxylum bungeanum* has a range of pharmacological effects such as antibacterial, anti-inflammatory, and antioxidant. However, there were no relevant studies on the regulation of gut microbes by EOZB in ruminants. In this study, the effects of different doses of EOZB on the structure and distribution of microorganisms in the small intestine of small-tailed Han sheep (STH) were investigated by 16s rRNA gene sequencing technique. We found that with the intervention of EOZB. The differential bacteria of duodenal at the phylum level were *Firmicutes*, *Bacteroidetes*, *Tenericutes* and *Proteobacteria*, and genus level differential bacteria were *Prevotella 1*, *Ruminococcus 2* and *Eubacterium coprostanoligenes group*. The differential bacteria of jejunal at the phylum level were *Firmicutes*, *Bacteroidetes*, *Tenericutes* and *Proteobacteria*, and genus level differential bacteria were *Prevotella 1*, *Rikenellaceae RC9 gut group*, *Christensenellaceae R-7 group*, *Ruminococcaceae UCG-014*, *Saccharofermentans*, *Ruminococcaceae NK4A214 group* and *Prevotellaceae UCG-001*. The differential bacteria of ileal at the phylum level were *Firmicutes*, *Bacteroidetes* and *Tenericutes*, and genus level differential bacteria were *Prevotella 1*, *Christensenellaceae R-7 group*, *Romboutsia* and *Ruminococcaceae UCG-014*. In addition, at the same dose of EOZB, the five most abundant genera of bacteria varied in different regions of the small intestine. Among them, the abundance of *Prevotella 1*, *Christensenellacea R-7 group* and *Ruminococcus 2* in ALW group was the highest in jejunum, duodenum and ileum, respectively. The abundance of *Prevotella 1*, *Christensenellacea R-7 group* and *Rikenellacea RC9 gut group* in BLW group was the highest in duodenum, jejunum and ileum, respectively. The abundance of *Prevotella 1*, *Christensenellacea R-7 group* and *Ruminococcaeae NK4A214 group* in CLW group was the highest in jejunum, duodenum and ileum, respectively. The abundance of *Prevotella 1*, *Ruminococcus 2* and *Ruminococcus NK4A214 groups* in DLW group was the highest in jejunum, duodenum and ileum, respectively. Differential bacteria formed under the regulation of EOZB are associated with the digestion and absorption of nutrients and the state of intestinal health in the host. This study is the first to investigate the effect of EOZB on the distribution and structure of bacteria in the small intestine of STH. The results of the study enriched the structure and distribution of bacteria in the small intestine of ruminants and provided new insights into the future application of herbal medicine in ruminant production. Additionally, it provides a theoretical basis for the selection of probiotic bacteria for ruminants and the development and application of microecological preparations.

## Introduction

Ruminants are essential for humans because they transform plant fibers into milk and meat (Eisler et al., 2014). In contrast to monogastric animals (such as pigs and poultry), ruminants have a compound stomach in the gastrointestinal tract (GIT) with four chambers: the rumen, reticulum, omasum, and abomasum. The numerous rumen microorganisms are essential in the processing of plant-based diets and produce precursors (such as volatile fatty acids) to provide ruminants with energy, as well as greenhouse gasses (such as methane; Russell and Rychlik, 2001; Moraïs and Mizrahi, 2019). Taxonomic profiles of rumen microorganisms and associated functions have been extensively investigated (Seshadri et al., 2018; Stewart et al., 2018, 2019; Li et al., 2020). The knowledge gained in previous studies has helped control rumen fermentation (Yáñez-Ruiz et al., 2015), increase feed effectiveness (Shabat et al., 2016), and reduce methane emissions (Shi et al., 2014), demonstrating that the GIT microbial community is crucial in rumen physiological processes (O'Hara et al., 2020). Additionally, the gut (GT) microbiota, commonly referred to as the “forgotten organ,” is a complex and varied community. The GT microbiota function as a bioinformatic hub that combines host metabolism, immunological functions, and genetic signals with outside inputs such as nutrition, medications, and probiotics (Thaiss et al., 2016). Anaerobic bacteria are prevalent in the mammalian distal gut where they break down nutrients such as polysaccharides (Backhed et al., 2005). The immune system and general health of hosts are substantially influenced by the large gut microbial community (Gillor et al., 2008; Schuijt et al., 2013). The ruminant digestive tract is divided into 10 compartments (the rumen, reticulum, omasum, abomasum, duodenum, jejunum, ileum, cecum, colon, and rectum), each compartment has a very diverse microbial composition and function. However, understanding of ruminant microbial processes is incomplete (Greening et al., 2019), even though the composition and function of microbial communities affect the ruminant digestive, immune, metabolic, and endocrine processes (Martinez-Guryn et al., 2019). Therefore, to completely comprehend the functions of the ruminant gastrointestinal microbiota, a detailed description of the bacteria in all gastrointestinal regions is required.

The plant *Zanthoxylum bungeanum* (Rutaceae) is currently widely distributed in most of China and some Southeast Asian countries. *Zanthoxylum bungeanum* is rich in flavonoids, primarily including rutin, quercetin, foeniculin, hyperin, and isoquercitrin (Li et al., 2001), and is currently frequently used as a flavoring agent and traditional Chinese herbal medicine because of its distinctive flavor and medicinal properties (Tezuka et al., 2001). The plant provides a range of pharmacological benefits, including anti-inflammatory (Zhang et al., 2017), antioxidant (Zhang et al., 2014), and antibacterial (Guleria et al., 2013) activities. The *Z. bungeanum* essential oil (EOZB) is a viscous liquid derived from plant husks with a distinct flavor that has pharmacological properties similar to those of *Z. bungeanum* (Zhang et al., 2022). Diarrhea is one of the common diseases in young ruminants and causes great problems to the development of animal husbandry. Diarrhea is usually accompanied by dysbiosis of the intestinal microbiota, damage to the intestinal mucosal barrier and inflammation (Karczewski et al., 2010), and EOZB has potential antibacterial, anti-inflammatory and antioxidant properties. However, most of the current ruminant studies have focused on the rumen and there are no reports on the regulation of EOZB on the intestinal bacteria in ruminants. Therefore, research in this area was needed for refinement.

Due to the incomplete study of the structure of ruminant gut bacterial composition, and whether EOZB affects the structure of the ruminant gut bacteria. We investigated the structure and distribution of bacteria communities in 60 STH small intestines (duodenum, jejunum, and ileum) samples under the EOZB intervention by 16s rRNA high-throughput sequencing. Aimed to supplement the effects of EOZB on ruminant small intestinal bacteria and the incomplete of ruminant gut bacteria.

## Materials and methods

### Experimental design and sampling

Twenty STH (male; 3-months-old; initial weight, 23.57 ± 4.61 kg, *p* > 0.05) were obtained from a farm in Dingxi, Gansu Province, China. Test sheep were housed in individual pens that were regularly disinfected and cleaned (once a week). The 20 experimental sheep were randomly divided into four groups and provided the diets shown in Table 1. The test groups were the following: ALW: basic diet (BD; without EOZB, nutritional level was basically the same as in other test groups); BLW: BD + EOZB at 5 ml/kg; CLW: BD + EOZB at 10 ml/kg; and DLW: BD + EOZB at 15 ml/kg. Different doses of EOZB were given daily to the different test groups (EOZB was added by spraying during feeding. The dose of EOZB was obtained with reference to Zhang et al. (2017) and adjusted to the actual situation). To distinguish samples from the small intestine sites of the duodenum, jejunum, and ileum, duodenal samples from ALW, BLW, CLW, and DLW groups were recorded as AD1-5, BD1-5, CD1-5, and DD1-5, respectively; jejunum samples were recorded as AJ1-5, BJ1-5, CJ1-5, and DJ1-5, respectively; and ileal samples were recorded as AI1-5, BI1-5, CI1-5, and DI1-5, respectively. The experiment was conducted for 52 days, and at the end, each group of test sheep was subjected to electric shock (head shock) and then slaughtered by means of neck bloodletting. Referring to *Anatomy and Histology and Embryology of Domestic Animals* ligation was performed at the division points of each part of the small intestinal tract, and the small intestinal tissue at each part was incised with a scalpel at the middle of the ligated segment. The contents of each part of the small intestine (duodenum, jejunum, and ileum) were collected in 1.5-ml sterile polypropylene tubes, immediately frozen in liquid nitrogen at −80°C, and stored for analysis of bacterial communities.

**Table 1:** Experimental diet composition and nutritional level (DM basis) %.

Dietary composition	Contents	Nutritional levels^②^	Contents
Wheat straw	50	DE, MJ/kg	12.30
Concentrate pellets^①^	50	CP	11.62
		EE	5.06
		Ash	16.58
		Ga	1.32
		P	0.60
		NDF	61.89
Total	100	ADF	13.50

① Concentrated pellets were purchased from a manufacturer, and the percentage of each ingredient was as follows: corn, 45%; soybean meal, 20%; cottonseed meal, 10%; rapeseed meal, 10%; wheat bran, 10%; and premix, 5%. The premix provided the following per kg of diet: vitamin A, 15,000 IU; vitamin D, 5,000 IU; vitamin E, 50 mg; Fe, 80 mg; Cu, 10 mg; Mn, 40 mg; Zn, 80 mg; Se, 0.2 mg; Co, 0.5 mg. ② Digestible Energy (DE) was a calculated value, whereas the other values were measured.

### DNA extraction and high-throughput sequencing

Total DNA was extracted from ruminal samples using an E.Z.N.A.1 Stool DNA Kit (Omega Bio-Tek, Norcross, GA, United States) according to the manufacturer’s protocol. The V4-V5 region of the bacterial 16S ribosomal RNA gene was amplified by PCR (95°C for 5 min, followed by 30 cycles at 95°C for 30 s, 55°C for 30 s, and 72°C for 45 s, and a final extension at 72°C for 5 min) using primers 515F (5′-GTGCCAGCMGCCGCGG-3′) and 907R (5′-CCGTCAATTCMTTTRAGTTT-3′), with the bar code a six-base sequence unique to each sample. The PCR reactions were performed in 30 μl of a mixture containing 15 μl of 2 × Phanta Master Mix, 1 μl of each primer (10 μM), and 20 ng of template DNA. Amplicons were extracted from 2% agarose gels and purified using an AxyPrep DNA Gel Extraction Kit (Axy-gen Biosciences, Union City, CA, United States) according to the manufacturer’s instructions. Purified PCR products were quantified by Qubit13.0 (Life Invitrogen, CA, United States), and every 20 amplicons with different bar codes were mixed equally. The pooled DNA product was used to construct an Illumina paired-end library following the Illumina genomic DNA library preparation procedure. The amplicon library was then pair-end sequenced (2 × 250) on an Illumina Novaseq 6000 platform (Nanjing GenePioneer Co. Ltd., Nanjing, China) according to the standard protocol.

### Bioinformatics analysis

Raw data returned by the Illumina HiSeq sequencing platform were filtered using the software packages Pandaseq (Masella et al., 2012), PRINSEQ (Schmieder and Edwards, 2011), and Vsearch (Rognes et al., 2016; v2.15.0_linux_x86_64) to remove chimeras and obtain optimized sequences (tags). Operational taxonomic units (OTUs) were clustered using Vsearch, and the clustering similarity threshold was 97%. A self-developed Perl program was used for random rarefaction of the data of each sample (the number of rarefactions was the minimum number of sample sequences). To select the most abundant sequence of each OTU as the representative sequence, QIIME (v.1.9.1) was used. Then, the Uclust method was used to compare the representative sequence to the Silva rRNA database (release_132) and classify the OTU species. Based on the abundance and annotation information of OTUs, the proportion of sequences in each sample at different taxonomic levels was counted to assess sample species abundance and diversity. Alpha diversity was assessed by Observed species, Chao1, Shannon, and Simpson indices (Li et al., 2013) and by plotting the samples as species accumulation curves. Beta diversity analysis was used to compare differences in species diversity (microbial composition and structure) between different samples. Sample-level PCoA and Anosim analysis (Sakaki et al., 1994; Dubois et al., 2010; with grouping information) were conducted based on Bray–Curtis distance and Unweighted Unifrac distance, respectively. Analysis of significant differences between groups (LEfSe analysis) was used to identify biomarkers (Segata et al., 2011). To illustrate the composition of bacterial communities in all test groups, heat maps were constructed of genera in the duodenum, jejunum, and ileum of the small intestine.

### Statistical data analysis

One-way ANOVA was performed using SPSS software (v21.0, SPSS Inc.). Analyzes were conducted on relative abundances and alpha diversity indices of bacteria in different parts of the small intestine at different levels. Multiple comparisons between groups were performed using Duncan’s method. Results are expressed as the mean and SEM, with *p* < 0.05 indicating significant differences.

## Results

### Effects of *Zanthoxylum bungeanum* essential oil on duodenal bacteria

**Figure 1:**
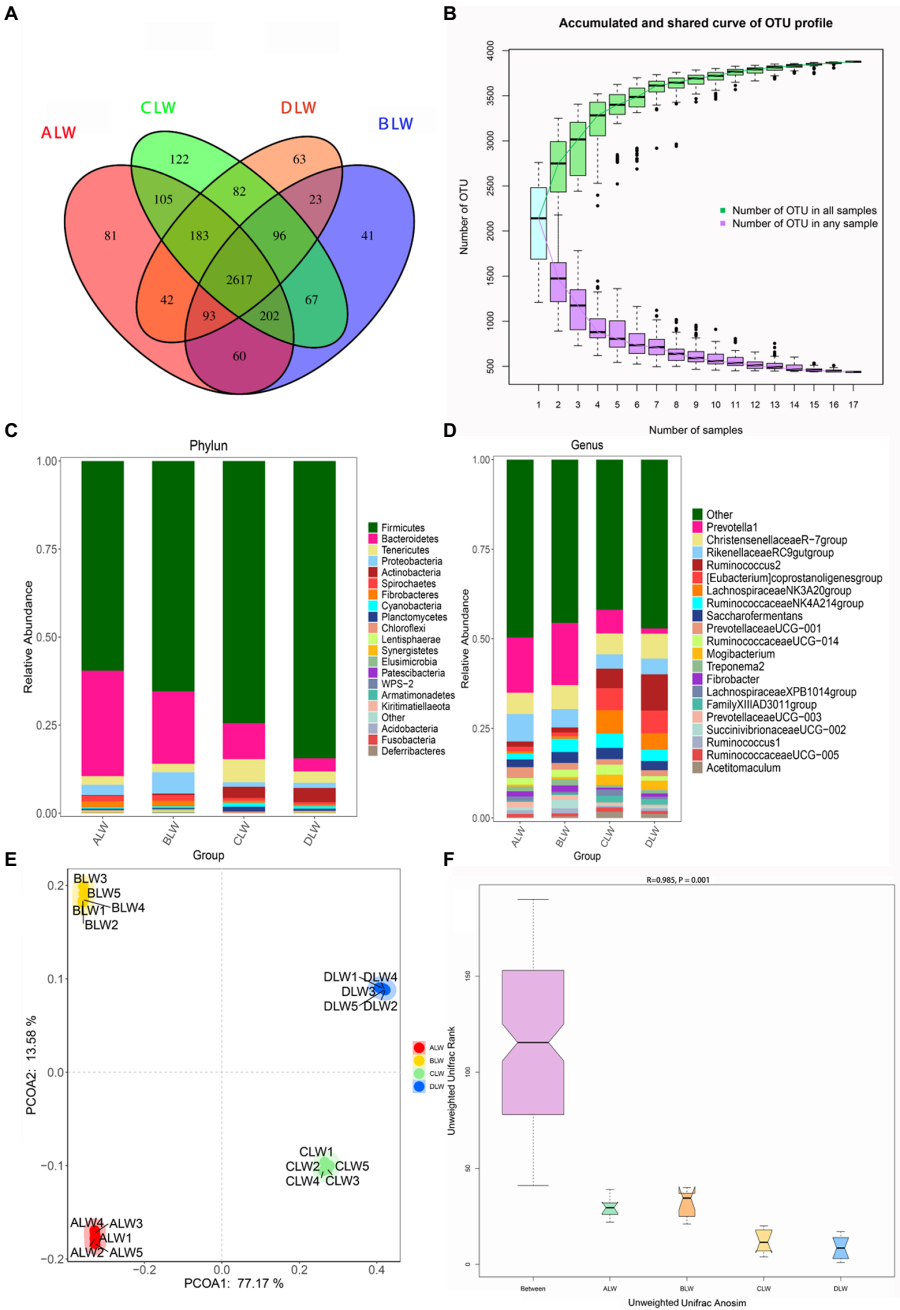
**(A)** OTU-Venn diagram **(B)** Species accumulation curves **(C,D)** Relative abundance of species at the phylum and genus level **(E,F)** PCoA and Anosim analysis.

A total of 1,992,846 tags were obtained from 20 duodenum samples; 1,873,850 clean tags were obtained after filtering, optimization, and quality control; and 78,484 sequences were extracted from each sample for subsequent analysis based on the minimum value of detected sequences (Supplementary Table S1). Clustering was performed using Vsearch with 97% similarity to obtain the OTUs of each sample. A total of 4,617 OTUs were generated, and 3,877 were obtained after random rarefaction, with representative OTU sequences selected for species annotation. There were 2,617 shared OTUs in the four groups, whereas 81 OTUs were specific to ALW, 41 to BLW, 122 to CLW, and 63 to DLW (Figure 1A). Species accumulation curves, widely used to determine the adequacy of sample size and to estimate species richness, were used to describe the increase in species with increasing sample size. The curve began to plateau at the sample size of 10, indicating that the sample size was sufficient and that the sequencing coverage was saturated (Figure 1B).

The dominant phyla of bacteria were *Firmicutes*, *Bacteroidetes*, *Tenericutes*, and *Proteobacteria*, which had relative abundances >1% in all groups (Figure 1C). Relative abundance of *Firmicutes* was significantly higher in DLW than in the other groups (*p* < 0.001), whereas that of *Bacteroidetes* was significantly higher in ALW than in the other groups (*p* < 0.001). Relative abundance of *Tenericutes* was significantly higher in CLW than in the other groups (*p* = 0.034), whereas that of *Proteobacteria* was significantly higher in BLW than in the other groups (*p* = 0.008; Supplementary Table S2). The dominant genera were *Prevotella 1*, *Christensenellaceae R-7 group*, *Ruminococcus 2*, *Rikenellaceae RC9 gut group*, *Eubacterium coprostanoligenes group*, *Ruminococcaceae NK4A214 group*, *Saccharofermentans*, *Ruminococcaceae UCG-014*, and *Prevotellaceae UCG-001*, which had relative abundances >1% in each test group (Figure 1D). Relative abundance of *Prevotella 1* was significantly higher in BLW than in the other groups (*p* < 0.001); whereas relative abundances of *Ruminococcus 2* and *Eubacterium coprostanoligenes group* were significantly higher in DLW than in the other groups (*p* < 0.001 and *p* = 0.001, respectively; Supplementary Table S2).

**Figure 2:**
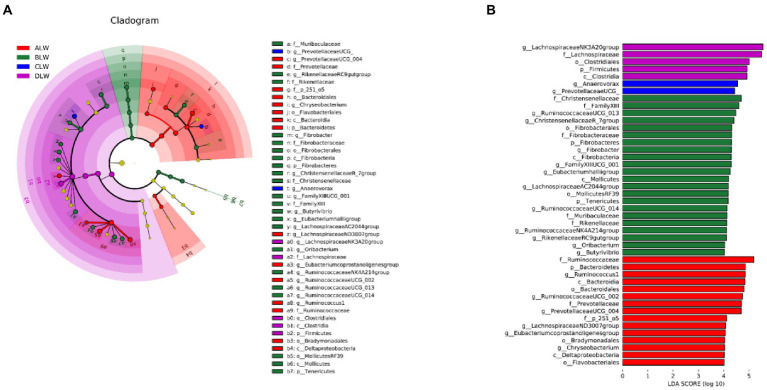
**(A)** Evolution cladogram **(B)** LDA score chart, LDA value >4, *p* < 0.05.

Principal coordinate analysis (PCoA) based on Bray–Curtis distance showed significant differences in bacterial taxa among the groups (Figure 1E). Anosim analysis based on Unweighted Unifrac distance further showed that the between-group differences were significantly greater than the within-group differences (Figure 1F). The Chao1 index, an indicator of community richness, was significantly higher in ALW than in the other groups (*p* < 0.001; Table 2). The LEfSe analysis showed that the addition of EOZB produced a total of 44 biomarkers (LDA score > 4; *p* < 0.05; Figures 2A,B).

**Table 2:** Effect of EOZB on duodenal alpha diversity index.

Index	ALW	BLW	CLW	DLW	SEM	*p*-value
Chao1	2372.76a	2027.55b	1830.25c	1642.20d	16.61	<0.001
Observed_species	2344.60	1933.60	1929.80	1714.20	23.07	0.201
Shannon	8.90	8.38	8.09	7.42	0.56	0.102
Simpson	0.99	0.99	0.98	0.98	0.01	0.301

### Effect of *Zanthoxylum bungeanum* essential oil on jejunal bacteria

**Figure 3:**
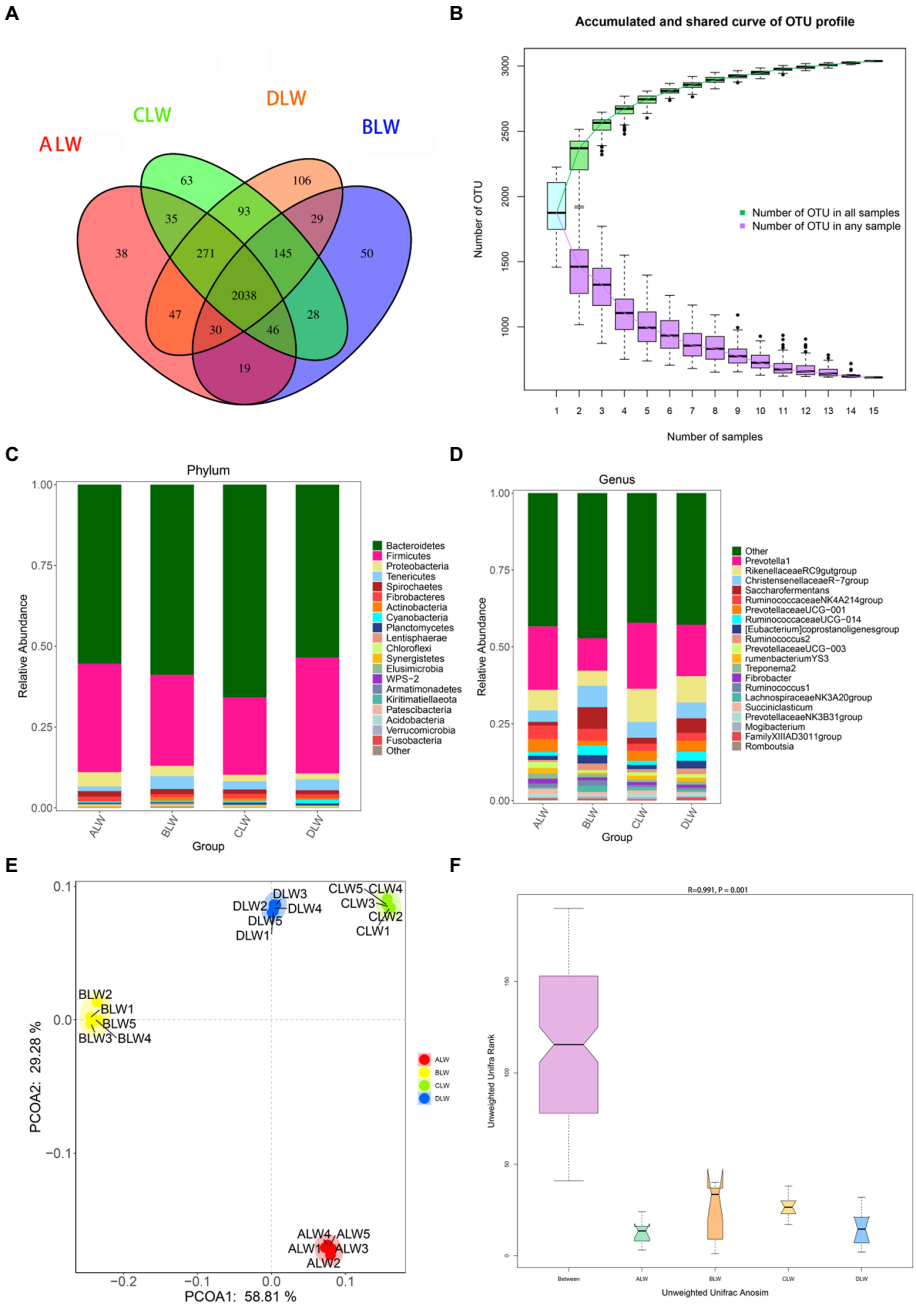
**(A)** OTU-Venn diagram **(B)** Species accumulation curves **(C,D)** Relative abundance of species at the phylum and genus level **(E,F)** PCoA and Anosim analysis.

A total of 1,783,198 tags were obtained from 20 jejunum samples; 1,652,166 clean tags were obtained after filtering, optimization, and quality control; and 72,109 sequences were extracted from each sample for subsequent analysis based on the minimum value of detected sequences (Supplementary Table S3). Clustering was performed using Vsearch with 97% similarity to obtain the OTUs of each sample. A total of 3,776 OTUs were generated, and 3,038 were obtained after random rarefaction, with representative OTU sequences selected for species annotation. There were 2,038 shared OTUs in the four groups, whereas 38 OTUs were specific to ALW, 50 to BLW, 63 to CLW, and 106 to DLW (Figure 3A). The species accumulation curve began to plateau at the sample size of 10, indicating that the sample size was sufficient and that the sequencing coverage was saturated (Figure 3B).

The dominant phyla of bacteria were *Firmicutes*, *Bacteroidetes*, *Tenericutes*, *Proteobacteria*, and *Spirochaetes*, which had relative abundances >1% (Figure 3C). Compared with other groups, relative abundance of *Firmicutes* was significantly higher in DLW (*p* < 0.001); that of Bacteroidetes was significantly higher in CLW (*p* < 0.001); that of *Tenericutes* was significantly higher in BLW (*p* = 0.002); and that of *Proteobacteria* was significantly higher in ALW (*p* = 0.001; Supplementary Table S4). The dominant genera of bacteria were *Prevotella 1*, *Rikenellaceae RC9 gut group*, *Christensenellaceae R-7 group*, *Saccharofermentans*, *Ruminococcaceae NK4A214 group*, *Prevotellaceae UCG-001*, *Eubacterium coprostanoligenes group*, and *Ruminococcaceae UCG-014*, which had relative abundances >1% (Figure 3D). Compared with other groups, relative abundances of *Prevotella 1* and *Rikenellaceae RC9 gut group* were significantly higher in CLW (both *p* < 0.001), those of *Christensenellaceae R-7 group*, *Ruminococcaceae UCG-014*, and *Saccharofermentans* were significantly higher in BLW (*p* = 0.006, *p* = 0.049, and *p* < 0.001, respectively), and those of *Ruminococcaceae NK4A214 group* and *Prevotellaceae UCG-001* were significantly higher in ALW (*p* < 0.001 and *p* = 0.02, respectively; Supplementary Table S4).

**Figure 4:**
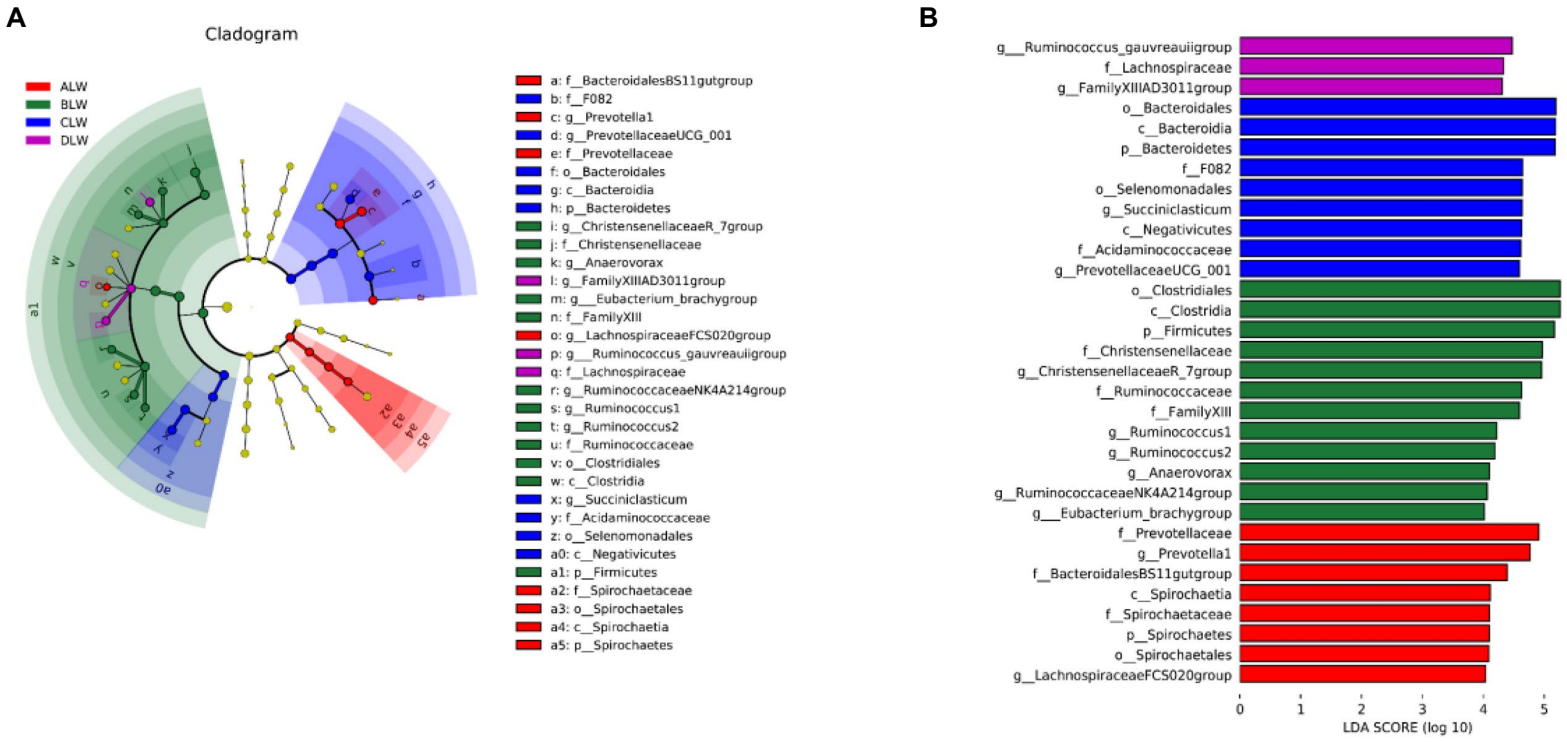
**(A)** Evolution cladogram **(B)** LDA score chart, LDA value >4, *p* < 0.05.

PCoA analysis showed significant differences in bacterial taxa among the groups (Figure 3E). According to the Anosim analysis, between-group differences were significantly greater than within-group differences (Figure 3F). The Chao1 index of community richness was significantly higher in DLW than in the other groups (*p* < 0.001; Table 3). The LEfSe analysis showed that the addition of EOZB produced a total of 32 biomarkers (LDA score > 4; *p* < 0.05; Figures 4A,B).

**Table 3:** The effect of EOZB on jejunum alpha diversity index.

Index	ALW	BLW	CLW	DLW	SEM	*p*-value
Chao1	2106.62c	1945.18d	2350.20b	2444.30a	7.83	<0.001
Observed_species	1897.00	1777.20	1972.60	2013.40	137.67	0.364
Shannon	8.77	8.35	8.76	8.62	0.28	0.426
Simpson	0.99	0.99	0.99	0.99	0.01	0.543

### Effect of *Zanthoxylum bungeanum* essential oil on Ileal bacteria

**Figure 5:**
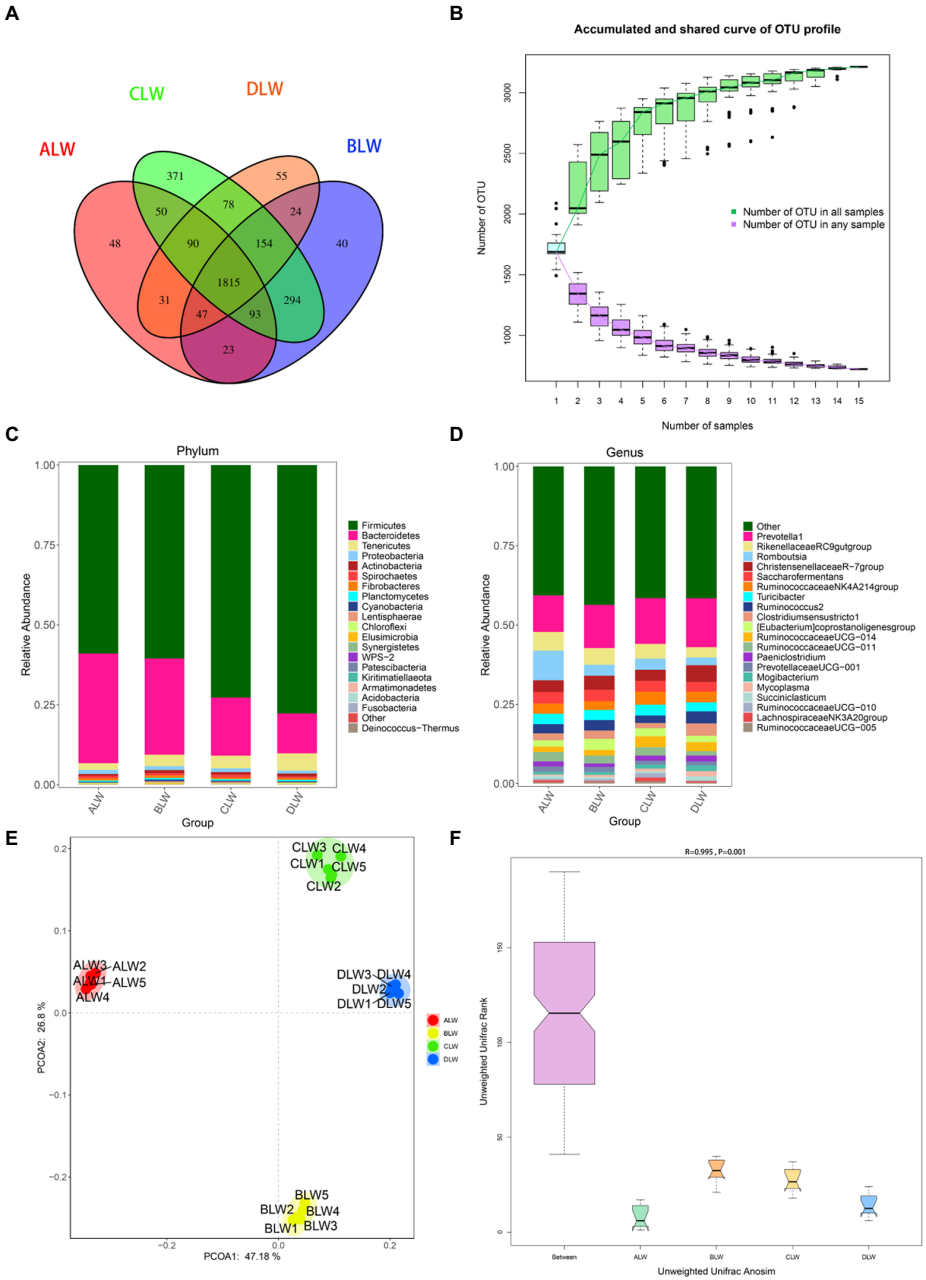
**(A)** OTU-Venn diagram **(B)** Species accumulation curves **(C,D)** Relative abundance of species at the phylum and genus level **(E,F)** PCoA and Anosim analysis.

A total of 1,551,651 tags were obtained from 20 ileum samples; 1,470,809 clean tags were obtained after filtering, optimization, and quality control; and 62,344 sequences were extracted from each sample for subsequent analysis based on the minimum value of detected sequences (Supplementary Table S5). Clustering was performed with 97% similarity using Vsearch to obtain the OTUs of each sample. A total of 3,944 OTUs were generated, and 3,213 were obtained after random rarefaction, with representative OTU sequences selected for species annotation. There were 1,815 shared OTUs in the four groups, whereas 48 OTUs were specific to ALW, 40 to BLW, 371 to CLW, and 55 to DLW (Figure 5A). The species accumulation curve began to plateau at the sample size of 10, indicating that the sample size was sufficient and that the sequencing coverage was saturated (Figure 5B).

The dominant phyla of bacteria were *Firmicutes*, *Bacteroidetes*, and *Tenericutes*, which had relative abundances >1% (Figure 5C). Compared with other groups, relative abundances of *Firmicutes* and *Tenericutes* were significantly higher in DLW (both *p* < 0.001) and that of *Bacteroidetes* was significantly higher in ALW (*p* < 0.001; Supplementary Table S6). At the genus level, *Prevotella 1*, *Romboutsia*, *Christensenellaceae R-7 group*, *Rikenellaceae RC9 gut group*, *Turicibacter*, *Ruminococcaceae NK4A214 group*, *Saccharofermentans*, *Clostridium sensu stricto 1*, *Ruminococcus 2*, *Eubacterium coprostanoligenes group*, *Ruminococcaceae UCG-011*, *Ruminococcaceae UCG-014*, *Paeniclostridium*, and *Prevotellaceae UCG-001* were the dominant bacteria, which had relative abundances >1% (Figure 5D). Compared with other groups, relative abundances of *Prevotella 1* and *Christensenellaceae R-7 group* were significantly higher in DLW (*p* < 0.001 and *p* = 0.001, respectively), that of *Romboutsia* was significantly higher in ALW (*p* < 0.001), and that of *Ruminococcaceae UCG-014* was significantly higher in CLW (*p* = 0.039; Supplementary Table S6).

**Figure 6:**
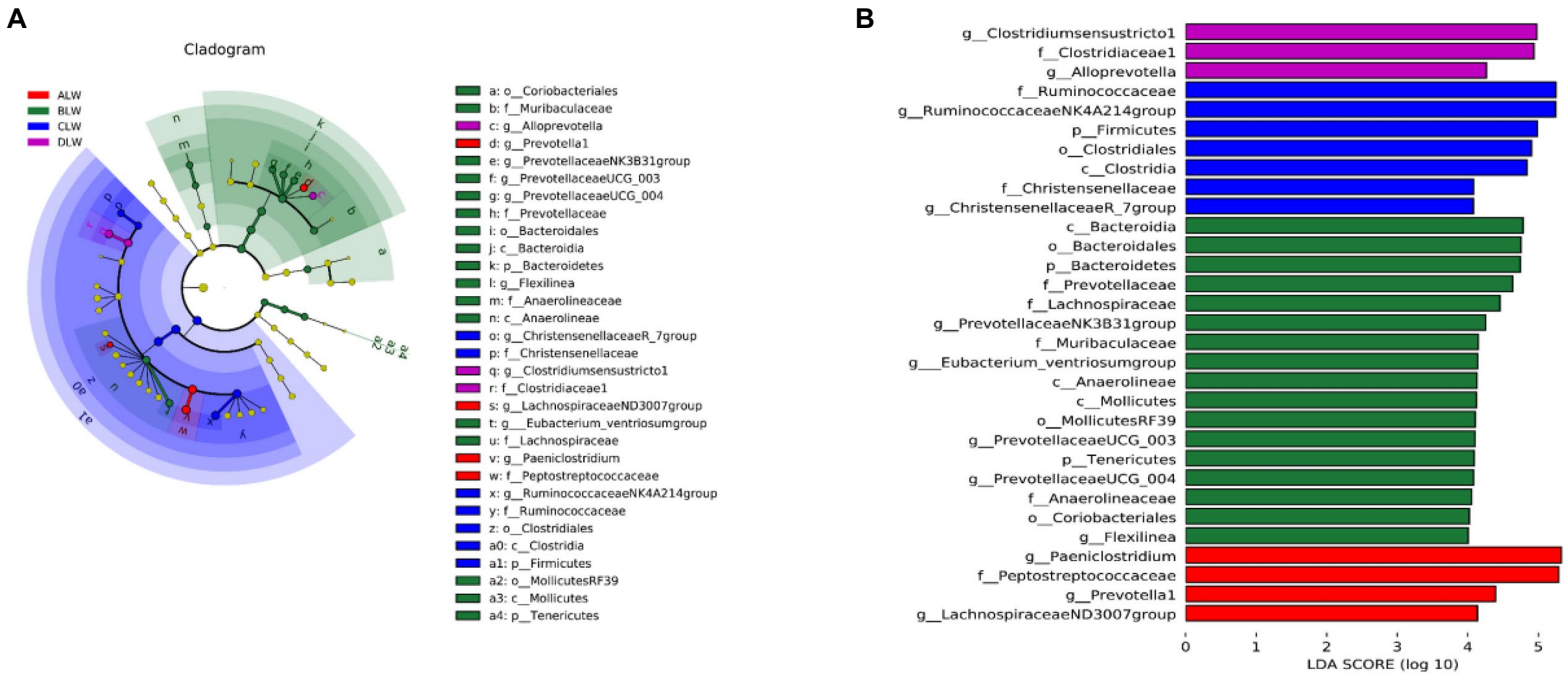
**(A)** Evolution cladogram **(B)** LDA score chart, LDA value >4, *p* < 0.05.

PCoA analysis showed significant differences in bacterial taxa among the test groups (Figure 5E). According to the Anosim analysis, between-group differences were significantly greater than within-group differences (Figure 5F). The Chao1 index of community richness was significantly higher in CLW than in the other groups (*p* < 0.001; Table 4). The LEfSe analysis showed that the addition of EOZB produced a total of 31 biomarkers (LDA score > 4; *p* < 0.05; Figures 6A,B).

**Table 4:** The effect of EOZB on ileum alpha diversity index.

Index	ALW	BLW	CLW	DLW	SEM	*p*-value
Chao1	1956.32c	2038.33b	2253.41a	2044.37b	13.53	<0.001
Observed_species	1699.40	1699.60	1760.80	1787.20	35.35	0.054
Shannon	8.27	8.32	8.25	8.26	0.05	0.485
Simpson	0.98	0.99	0.99	0.98	0.01	0.519

### Effect of *Zanthoxylum bungeanum* essential oil on bacteria in different sites of the small intestine

To further understand effects of EOZB on bacteria in the small intestine, a heat map was constructed for all genera (at the same dose of EOZB) in different sites of the small intestine.

**Figure 7:**
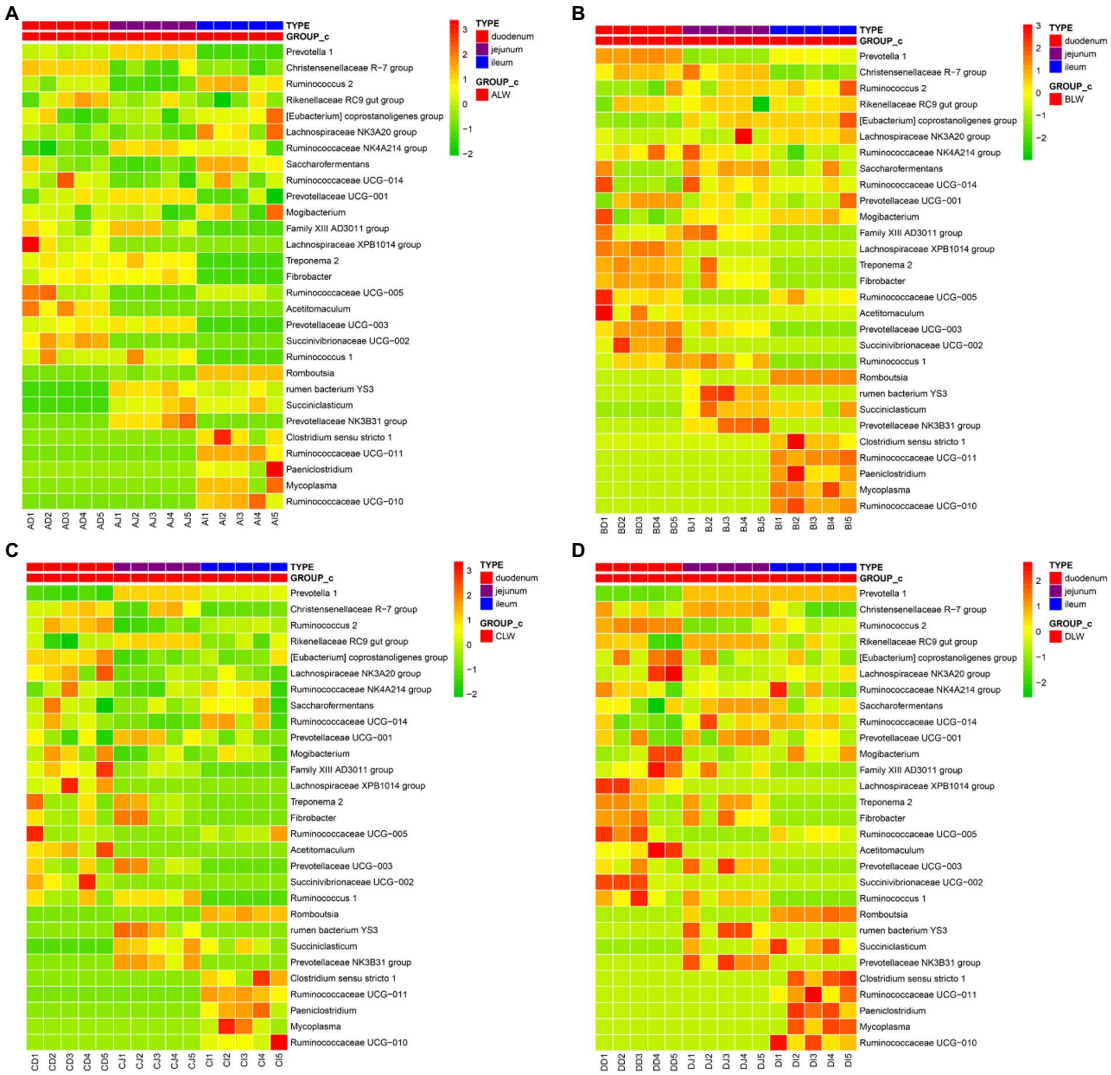
**(A)** Heat map of microbial distribution at the genus level in the ALW group **(B)** Heat map of microbial distribution at the genus level in the BLW group **(C)** Heat map of microbial distribution at the genus level in the CLW group **(D)** Heat map of microbial distribution at the genus level in the DLW group.

The five most abundant genera in the duodenum of the ALW group were *Prevotella 1*, *Rikenellaceae RC9 gut group*, *Christensenellaceae R-7 group*, *Prevotellaceae UCG-001*, and *Saccharofermentans*. In the jejunum, the five most abundant genera were *Prevotella 1*, *Rikenellaceae RC9 gut group*, *Ruminococcaceae NK4A214 group*, *Prevotellaceae UCG-001*, and *Christensenellaceae R-7 group*. In the ileum, the five most abundant genera were *Prevotella 1*, *Romboutsia*, *Rikenellaceae RC9 gut group*, *Christensenellaceae R-7 group*, and *Saccharofermentans*. In addition, the same genus of bacteria was distributed differently in different parts of the small intestine. The highest abundance of *Prevotella 1* was in the jejunum, that of *Christensenellaceae R-7 group* was in the duodenum, and that of *Ruminococcus 2* was in the ileum (Figure 7A).

The five most abundant genera of bacteria in the duodenum of the BLW group were *Prevotella 1*, *Christensenellaceae R-7 group*, *Rikenellaceae RC9 gut group*, *Ruminococcaceae NK4A214 group*, and *Saccharofermentans*. In the jejunum, the five most abundant genera were *Prevotella 1*, *Saccharofermentans*, *Christensenellaceae R-7 group*, *Rikenellaceae RC9 gut group*, and *Ruminococcaceae NK4A214 group*. In the ileum, the five most abundant genera were *Prevotella 1*, *Rikenellaceae RC9 gut group*, *Christensenellaceae R-7 group*, *Saccharofermentans*, and *Eubacterium coprostanoligenes group*. In addition, the same genus of bacteria was distributed differently in different parts of the small intestine. The highest abundance of *Prevotella 1* was in the duodenum, that of *Christensenellaceae R-7 group* was in the jejunum, and that of *Rikenellaceae RC9 gut group* was in the ileum (Figure 7B).

The five most abundant genera in the duodenum of the CLW group were *Prevotella 1*, *Lachnospiraceae NK3A20 group*, *Eubacterium coprostanoligenes group*, *Christensenellaceae R-7 group*, and *Ruminococcus 2*. In the jejunum, the five most abundant genera were *Prevotella 1*, *Rikenellaceae RC9 gut group*, *Christensenellaceae R-7 group*, *Prevotellaceae UCG-001*, and *Ruminococcaceae NK4A214 group*. In the ileum, the five most abundant genera were *Prevotella 1*, *Rikenellaceae RC9 gut group*, *Christensenellaceae R-7 group*, *Saccharofermentans*, and *Ruminococcaceae UCG-014*. In addition, the same genus of bacteria was distributed differently in different parts of the small intestine. The highest abundance of *Prevotella 1* was in the jejunum, that of *Christensenellaceae R-7 group* was in the duodenum, and that of *Ruminococcaceae NK4A214 group* was in the ileum (Figure 7C).

The five most abundant genera in the duodenum of the DLW group were *Ruminococcus 2*, *Christensenellaceae R-7 group*, *Eubacterium coprostanoligenes group*, *Lachnospiraceae NK3A20 group*, and *Rikenellaceae RC9 gut group*. The five most abundant genera in the jejunum were *Prevotella 1*, *Christensenellaceae R-7 group*, *Rikenellaceae RC9 gut group*, *Saccharofermentans*, and *Prevotellaceae UCG-001*. The five most abundant genera in the ileum were *Prevotella 1*, *Christensenellaceae R-7 group*, *Clostridium sensu stricto 1*, *Ruminococcus 2*, and *Rikenellaceae RC9 gut group*. In addition, the same genus was distributed differently in different parts of the small intestine. The highest abundance of *Prevotella 1* was in the jejunum, that of *Ruminococcus 2* was in the duodenum, and that of *Ruminococcaceae NK4A214 group* was in the ileum (Figure 7D).

## Discussion

In this study, *Firmicutes*, *Bacteroidetes*, *Tenericutes*, and *Proteobacteria* were the dominant phyla of bacteria in the duodenum of STH. Compared with other trial groups, *Firmicutes*, *Tenericutes*, and *Proteobacteria* increased significantly in the DLW, CLW, and BLW groups (*p* < 0.001, *p* = 0.034, and *p* = 0.008). Interestingly, *Bacteroidetes* decreased significantly in the DLW groups (*p* < 0.001). *Firmicutes* primarily include many gram-positive bacteria, including *Lactococcus*, *Listeria*, *Bacillus*, and *Lactobacillus* (Garneau et al., 2008). *Lactobacillus*, *Listeria*, and *Lactococcus* are considered beneficial bacteria that were important in maintaining the balance of intestinal microbiota and preventing pathogenic invasion (Wang et al., 2018; Li et al., 2019). Furthermore, *Firmicutes* have key roles in the digestion of proteins and carbohydrates (Spence et al., 2006). The main function of *Bacteroidetes* was to increase the utilization of carbohydrates (Jami et al., 2013). In addition, a high F/B (*Firmicutes*/*Bacteroides*) ratio helps a host to absorb energy and maintain microbiota homeostasis (Fernando et al., 2010; Murphy et al., 2010). In this study, the addition of EOZB increased significantly the abundance of *Firmicutes* and decreased significantly the abundance of *Bacteroidetes* in the DLW group, which caused an increase in the F/B ratio. This may be due to the antimicrobial properties of EOZB, where many microorganisms competed with each other, and a decrease in the number of one part of the microorganisms in competition may lead to an increase in the number of another part. It also indicated that EOZB may have a role in improving energy absorption, maintaining microbiota homeostasis, maintaining intestinal microorganism balance, and improving protein and carbohydrate digestion. *Tenericutes* were associated with fat deposition in Angus bull muscle (Zhan et al., 2017; Krause et al., 2020), and *Proteobacteria* were associated with energy accumulation (Amato et al., 2014; Chevalier et al., 2015). We found that the addition of EOZB increased significantly the abundance of *Tenericutes* in the CLW group and increased significantly the abundance of *Proteobacteria* in the BLW group. This may be due to the dose of EOZB, and different microorganisms may have different sensitivity to the dose of EOZB. It also indicated that EOZB may have a role in regulating energy accumulation and fat deposition. The dominant genera in the duodenum of STH were *Prevotella 1*, *Christensenellaceae R-7 group*, *Ruminococcus 2*, *Rikenellaceae RC9 gut group*, *Eubacterium coprostanoligenes group*, *Ruminococcaceae NK4A214 group*, *Saccharofermentans*, *Ruminococcaceae UCG-014*, and *Prevotellaceae UCG-001*. Relative abundances of *Prevotella 1*, *Ruminococcus 2*, and *Eubacterium coprostanoligenes group* were significantly higher in BLW, DLW, and DLW than in the other group (*p* < 0.001, *p* < 0.001, and *p* = 0.001, respectively). *Prevotella 1* can degrade fiber-derived feeds such as hemicellulose or xylan (Emerson and Weimer, 2017). In addition, *Prevotella* can use starch, monosaccharides, and other noncellulosic polysaccharides (Purushe et al., 2010). *Ruminococcus 2* belongs to *Ruminococcus*, which primarily inhabits the rumen and hindgut of ruminants and contributes to the degradation of cellulose and starch (Zhao et al., 2018). *Ruminococcus* also produces acetic acid, formic acid, and a small amount of lactic acid (Berg Miller et al., 2009). Short-chain fatty acids, including formic acid and acetate, have key roles in regulating gut microbiota balance and maintaining the morphology and function of intestinal epithelial cells (Tan et al., 2014; Li et al., 2019). The *Eubacterium coprostanoligenes group* functions in converting cholesterol to coproitol, which can further affect the fat metabolism of a host (Freier et al., 1994). We found that supplementation of EOZB increased significantly the abundance of *Prevotella 1* in the BLW group, *Ruminococcus 2* and *Eubacterium coprostanoligenes group* in the DLW group. This may be due to the dose and antimicrobial properties of EOZB, with different microorganisms responding differently to different doses of EOZB. It also suggests that EOZB may have a role in maintaining intestinal microorganism balance and enhancing carbohydrate digestion and absorption with lipid metabolism. This was similar to the function of differential microbiota at the phylum level.

*Firmicutes*, *Bacteroidetes*, *Tenericutes*, *Proteobacteria*, and *Spirochaetes* were the dominant phyla of bacteria in the jejunum of STH. Relative abundances of *Firmicutes*, *Bacteroidetes*, *Tenericutes*, and *Proteobacteria* increased significantly in DLW, CLW, BLW, and ALW groups than in the other groups (*p* < 0.001, *p* < 0.001, *p* = 0.002, and *p* = 0.001, respectively). *Spirochaetes* were closely associated with fat metabolism pathways (Yang et al., 2022) and can degrade pectin and xylan (Liu et al., 2014; Tokuda et al., 2018). *Spirochaetes* was the dominant bacteria in this study, but the difference in relative abundance between groups was not significant. However, *Firmicutes*, *Bacteroidetes*, *Tenericutes* and *Proteobacteria* in the dominant bacteria increased significantly in the DLW, CLW, BLW and ALW groups than in the other trial groups. This may be due to jejunal function and the dose of EOZB, the jejunum of ruminants is dominated by chemical digestion and the main function of microorganisms is to secrete various digestive enzymes. Combined with the functions of the dominant bacteria mentioned earlier, this may indicate that EOZB has a role in regulating the microorganisms that secrete various digestive enzymes and thus improve carbohydrate and lipid absorption. At the genus level, *Prevotella 1*, *Rikenellaceae RC9 gut group*, *Christensenellaceae R-7 group*, *Saccharofermentans*, *Ruminococcaceae NK4A214 group*, *Prevotellaceae UCG-001*, *Eubacterium coprostanoligenes group*, and *Ruminococcaceae UCG-014* were the dominant bacteria in the jejunum of STH. Relative abundances of *Prevotella 1*, *Rikenellaceae RC9 gut group*, *Christensenellaceae R-7 group*, *Ruminococcaceae UCG-014*, *Saccharofermentans*, *Ruminococcaceae NK4A214 group*, and *Prevotellaceae UCG-001* were significantly higher in CLW, CLW, BLW, BLW, BLW, ALW, and ALW, respectively, than in the other groups (*p* < 0.001, *p* < 0.001, *p* = 0.006, *p* = 0.049, *p* < 0.001, *p* < 0.001, and *p* = 0.02, respectively). *Rikenellaceae RC9 gut group* belongs to *Rikenellaceae* and most can ferment unabsorbed polysaccharides in the host gut to produce short-chain fatty acids such as acetate, propionate, and butyrate (Su et al., 2014). *Saccharofermentans* are anaerobic gram-negative bacteria that convert glucose to acetate, lactate, and fumarate *in vivo* (Chen et al., 2010). We found that supplementation with EOZB significantly increased the relative abundance of *Saccharofermentans* in the BLW group and the *Rikenellaceae RC9 gut group* in the CLW group. This may be related to the dose of EOZB, with different microorganisms responding to different doses of EOZB. It also suggests that EOZB may have a role in improving polysaccharide absorption and VFA (Volatile Fatty Acid) production. *Christensenellaceae R-7 group* is in the *Firmicutes* (Waters and Ley, 2019) and has a key role in the decomposition of fibrous substances (Evans et al., 2011). *Ruminococcaceae UCG-014* and *Ruminococcaceae NK4A214 group* are in the family *Ruminococcaceae* and have crucial roles in fiber degradation and biohydrogenation (Gagen et al., 2015; Opdahl et al., 2018). In addition, the relative abundance of *Ruminococcaceae* increases in beef cattle with high feed efficiency (Li and Guan, 2017). We found that EOZB supplementation significantly increased the abundance of *Christensenellaceae R-7 group* and *Ruminococcaceae UCG-014* and significantly decreased the abundance of the *Ruminococcaceae NK4A214 group* in the BLW group. This may be due to the dose and antimicrobial characteristics of EOZB, with different microorganisms responding to different doses of EOZB. It also suggests that EOZB may have a role in improving fiber degradation and feed efficiency. *Prevotellaceae UCG-001* is a *Prevotella* strain that has an important role in the degradation of cellulose and xylan. *Prevotellaceae UCG-001* was significantly enriched after feeding mice with inulin and thereby alleviated glucose and lipid metabolism disorders (Song et al., 2019). In addition, the proportion of *Prevotellaceae UCG-001* increases in the azomethane-induced mouse colon cancer model (Ibrahim et al., 2019). We found that *Prevotellaceae UCG-001* was significantly increased in the ALW group than in the other trail groups. This may be due to the anti-inflammatory characteristics of EOZB. EOZB has an anti-inflammatory effect, which in turn inhibits the growth of microorganisms associated with pro-inflammatory responses. It also suggests that EOZB may have a role in regulating the catabolism of fibrous substances, glycolipid metabolism and improving the inflammatory process.

*Firmicutes*, *Bacteroidetes*, and *Tenericutes* were the dominant phyla of bacteria in the ileum of STH. Relative abundances of *Firmicutes*, *Bacteroidetes*, and *Tenericutes* were significantly higher in DLW, ALW, and DLW, respectively than in the other groups (all *p* < 0.001). The functions of those phyla of bacteria are discussed in the section on the duodenum. At the genus level, *Prevotella 1*, *Romboutsia*, *Christensenellaceae R-7 group*, *Rikenellaceae RC9 gut group*, *Turicibacter*, *Ruminococcaceae NK4A214 group*, *Saccharofermentans*, *Clostridium sensu stricto 1*, *Ruminococcus 2*, *Eubacterium coprostanoligenes group*, *Ruminococcaceae UCG-011*, *Ruminococcaceae UCG-014*, *Paeniclostridium*, and *Prevotellaceae UCG-001* were the dominant bacteria in the ileum of STH. Relative abundances of *Prevotella 1*, *Christensenellaceae R-7 group*, *Romboutsia*, and *Ruminococcaceae UCG-014* were significantly higher in DLW, DLW, ALW, and CLW, respectively, than in the other groups (*p* < 0.001, *p* = 0.001, *p* < 0.001, and *p* = 0.039). *Romboutsia* is significantly positively correlated with total carbohydrate digestibility (Li et al., 2022). In addition, *Romboutsia* has effects on the inflammatory process and is inversely related to inflammatory bowel disease (Schirmer et al., 2019). We found that *Romboutsia* was significantly increased in the ALW group than the other trial groups. This may be due to the anti-inflammatory characteristics of EOZB, which reduces the relative abundance of *Romboutsia* by inhibiting the inflammatory process and thus. It also suggests that EOZB may have a role in regulating carbohydrate digestion and inflammatory processes.

At the same dose of EOZB, the composition of the five most abundant genera of bacteria differed in different sites of the small intestine. In the ALW group, the *Ruminococcaceae NK4A214 group* was a jejunum-specific genus of bacteria, whereas *Romboutsia* was an ileum-specific genus of bacteria. The *Ruminococcaceae NK4A214 group* is in the family *Ruminococcaceae* and is highly associated with biohydrogenation in ruminants, in addition to its role in fiber degradation, which in turn affects lipid metabolism (Wang et al., 2019). The genus *Romboutsia* is more abundant in healthy guts than in polyp-associated gut tissue, suggesting that *Romboutsia* is important in maintaining gut health (Mangifesta et al., 2018). This may be due to the function of different small intestinal sites, which have different functions and therefore different compositions of microorganisms that maintain these functions. It also suggests that lipid metabolism may be one of the functions of the jejunum and that the function of the ileum may be related to intestinal health. Among the most abundant genera of bacteria in the BLW group, *Eubacterium coprostanoligenes group* was the ileum-specific genus of bacteria. Compared with patients with irritable bowel syndrome, the *Eubacterium coprostanoligenes group* is the dominant bacteria in healthy controls (Liu et al., 2021). This may be due to the function of different parts of the small intestine and the anti-inflammatory characteristics of EOZB, with differences in function resulting in different microbial compositions. It also suggests that the function of the ileum may be related to the regulation of inflammation and that EOZB may have a role in regulating the inflammatory process. Among the most abundant genera of bacteria in the CLW group, *Lachnospiraceae NK3A20 group* was the duodenum-specific genus of bacteria. *Lachnospiraceae NK3A20 group* is in the family *Lachnospiraceae*, which is one of the main components of ruminant gastrointestinal microbiota (Kittelmann et al., 2013) and is closely associated with butyrate production (Vital et al., 2014; Haas and Blanchard, 2017). Butyrate contributes to the development of gastrointestinal epithelial cells in ruminants (Xu et al., 2001). This may be related to the anti-inflammatory characteristics of EOZB, where intestinal epithelial cells are part of non-specific immunity and the proliferation of epithelial cells can reduce the permeability of intestinal epithelial tissue to some extent, and the reduced permeability leads to a decrease in the passage of pro-inflammatory factors and pathogenic microorganisms, which in turn regulates the inflammatory process. It also suggests that the anti-inflammatory effect of EOZB may have a relationship with an intestinal barrier function. Among the most abundant genera of bacteria in the DLW group, *Ruminococcus 2* and *Lachnospiraceae NK3A20 group* were duodenal endemic bacteria, and *Clostridium sensu stricto 1* was the ileal endemic bacteria. *Clostridium sensu stricto 1* is a butyrate-producing genus of clostridia bacteria, and butyrate is essential for the development of gastrointestinal epithelial cells and as the final metabolite of unabsorbed polysaccharides by the gut microbiota, for host energy supply (Pryde et al., 2002). The results were similar to those of the CLW group, which may be related to the anti-inflammatory properties of EOZB. It also suggests that EOZB may have some connection with the production of butyrate. In addition, our other study also showed that EOZB significantly increased final weight, average daily gain, dry matter intake and feed conversion ratio in STH. This suggests that these bacteria that were altered by the intervention of EOZB may have a positive effect on the productive performance of STH.

## Conclusion

In this study, the affection of EOZB on bacterial distribution in the small intestine of STH was characterized. There were significant differences in the horizontal distribution of bacteria in the same part of the small intestine at different doses of EOZB. With EOZB, in the duodenum, there were four different phyla of bacteria and three different genera, whereas in the jejunum, there were four different phyla of bacteria and seven different genera. In the ileum, there were three different phyla and four different genera. The composition (vertical) of the five most abundant genera of bacteria was different in different parts of the small intestine (duodenum, jejunum, and ileum) at the same dose of EOZB. Some genera were specific to different sites in the small intestine at the same dose of EOZB. The differential genera of bacteria are associated with the digestion and absorption of host proteins, carbohydrates, and lipids. Simultaneously, those bacteria can have positive effects on the development of host intestinal epithelial cells, maintenance of microbial and metabolic homeostasis, and control of inflammatory processes. Thus, although the evidence is indirect, it indicates that EOZB affects the above physiological processes. Our findings fill the gap of bacteria composition structure of small intestine in ruminants, and also provide some theoretical basis for the selection of probiotic bacteria and the development and application of EOZB.

## Data availability statement

The datasets presented in this study can be found in online repositories. The names of the repository/repositories and accession number(s) can be found in the article/Supplementary material.

## Ethics statement

The animal study was reviewed and approved by Experimental Animal Ethics Committee of Gansu Agricultural University (Approval No. GSAU-Eth-AST-2021-026). Written informed consent was obtained from the owners for the participation of their animals in this study.

## Author contributions

CW and XL conceived, designed, and supervised the project. HZ, XL, and YZ collected samples and performed experiments. HZ and XL carried out bioinformatic analyzes. HZ drafted the manuscript. HZ, XL, YZ, and CW revised the manuscript. XL, HZ, and CW, contributed ideas on the paper. All authors have read, edited, and approved the final manuscript.

## Funding

This work was supported by the Project of Research Condition Construction and Achievement Transformation of Gansu Academy of Agricultural Sciences (Modern Biological Breeding, 2021GAAS01).

## Conflict of interest

The authors declare that the research was conducted in the absence of any commercial or financial relationships that could be construed as a potential conflict of interest.

## Publisher’s note

All claims expressed in this article are solely those of the authors and do not necessarily represent those of their affiliated organizations, or those of the publisher, the editors and the reviewers. Any product that may be evaluated in this article, or claim that may be made by its manufacturer, is not guaranteed or endorsed by the publisher.
